# Malaria knowledge and its associated factors among pregnant women attending antenatal clinic of Adis Zemen Hospital, North-western Ethiopia, 2018

**DOI:** 10.1371/journal.pone.0210221

**Published:** 2019-01-10

**Authors:** Yitayal Ayalew Goshu, Azeb Ewinetu Yitayew

**Affiliations:** Department of Midwifery, College of Health Sciences, Debre Tabor University, Debre, Tabor, Ethiopia; Bielefeld University, GERMANY

## Abstract

**Introduction:**

In Ethiopia, the burden of malaria during pregnancy remains a public health problem. Having a good malaria knowledge leads to practicing the prevention of malaria and seeking a health care. Researches regarding pregnant women’s knowledge on malaria in Ethiopia is limited. So the aim of this study was to assess malaria knowledge and its associated factors among pregnant woman, 2018.

**Methods:**

An institutional-basedcross-sectional study was conducted in Adis Zemen Hospital. Data were collected using pre-tested, an interviewer-administered structured questionnaire among 236 mothers. Women’s knowledge on malaria was measured using six malaria-related questions (cause of malaria, mode of transmission, signs and symptoms, complication and prevention of malaria). The collected data were entered using Epidata version 3.1 and exported to SPSS version 20 for analysis. Bivariate and multivariate logistic regressions were computed to identify predictor variables at 95% confidence interval. Variables having P value of <0.05 were considered as predictor variables of malaria knowledge.

**Result:**

A total of 235 pregnant women participated which makes the response rate 99.6%. One hundred seventy two pregnant women (73.2%) of mothers had good knowledge on malaria.Women who were from urban (AOR; 2.4: CI; 1.8, 5.7), had better family monthly income (AOR; 3.4: CI; 2.7, 3.8), attended education (AOR; 1.8: CI; 1.4, 3.5) were more knowledgeable.

**Conclusion and recommendation:**

Majority of participants had good knowledge on malaria. Educational status, household monthly income and residence werepredictors of malaria knowledge. Increasing women’s knowledge especially for those who are from rural, have no education, and have low monthly income is still needed.

## Introduction

Malaria is a life-threatening disease caused by parasites that is transmitted to people through the bites of infected female Anopheles mosquitoes [1]. Around half of the world's population is at risk of malaria but most malaria cases and deaths occur in sub-Saharan Africa. Despite malaria infection can affect all ages and sexes, morbidity and mortality related to malaria are very common in pregnant women and children less than five years of age [2,3]. According to Center for Disease Control (CDC), pregnant women lose some of their immunity and are prone to malaria infection because of the changes in women’s immune systems during pregnancy and the presence of placenta with new places for parasites to bind [4,5].

Despite the incidence of malaria and deaths due to malaria from 1995 to 2015 decreased in Ethiopia, the morbidity and mortality of the population due to malaria is still a major health problem that needs to be solved timely [6]. As malaria is preventable and curable, increased malaria prevention and control measures are dramatically reducing the malaria burden in many places[7].

Additionally; malaria causes significant economic losses, and can decrease the gross domestic product (GDP) by as much as 1.3% in countries with high levels of transmission [4]. In the long run,there will be substantial differences in GDP between countries with and without malaria, particularly in Africa [2, 3]. According to WHO, in some heavy-burden countries, the disease accounts for up to 40% of public health expenditures, 30% to 50% of inpatient hospital admissions, up to 60% of outpatient health clinic visits [1, 5].

Moreover; malaria infection during pregnancy remains a preventable cause of maternal mortality and morbidity globally, including in Ethiopia [8]. According to EDHS 2016, maternal mortality is a major public health problem which shows that the maternal mortality ratio is 412 per 100, 000 live births and malaria contributes as a cause for maternal mortality [8,9]. Malaria infection during pregnancy can have adverse effects on both the mother and fetus, including maternal anemia, maternal mortality, fetal loss, premature delivery, intrauterine growth retardation, and delivery of low birth-weight infants (<2500 g or <5.5 pounds), a risk factor for death which implies a major health problem for newborn and mother [10–13].

Every year in Sub-Saharan Africa there are about 25 million pregnancies which are at risk for malaria infection[14, 15]. In Ethiopia, the burden of malaria during pregnancy remains a public health importance, including its complication for the newborn and mother. Studies done in southern Ethiopian and northwestern Ethiopia revealed that the prevalence of malaria among pregnant women was 9.7 and 10.4% respectively [16, 17].

Studies done in Sub-Saharan Africa including Ethiopia showed that women’s knowledge regarding malaria remains low [18–23]. Studies done in Nigeria [18], BurkinFaso[19], and Sudan [20] revealed that 64.9%, 56.1% and 55.9% of participants had good knowledge on malaria respectively.

Cross-sectional studies were done in Ethiopia especially in Shahsango [24], Bonke [25], and Tepi [26] to assess knowledge on malaria among pregnant women showed that 74.1%, 16.5%and 17.7% of the respondents had good knowledge respectively. However; different studies reveal that practice of malaria preventive measure and health-seeking behavior of the community are related to the level of knowledge. Having a good knowledge regarding malaria cause, mode of transmission, sign and symptom, the effect of malaria on pregnancy and prevention of malaria leads to use malaria prevention mechanism and increase health-seeking behavior [23–24, 27–30]. In Ethiopia, women’s level of practice on malaria preventive measure is too poor [23,24,26,29]. Therefore, determining women’s knowledge on malaria is an important solution.

Different articles revealed that knowledge of pregnant women on malaria is influenced by socio-demographic characteristics like education status, occupation, residence, ownership of television or radio, religion, ethnicity, age, and family monthly income[18–23].

As there is a high burden of malaria, low knowledge of malaria and studies done in Ethiopia are limited; this study was conducted in Adis Zemen primary hospital to assess knowledge on malaria and its associated factors of among pregnant woman. So this study will be helpful in guiding policymakers and concerned bodies and will be used as baseline information for other investigators.

## Methods and materials

### Setting

This institutional-based cross-sectional study was carried out in Adis Zemen primary hospital from May1-30, 2018. Addis Zemen primary hospital is found in Adis Zemen town which is an administrative town of Libo Kemkem Wereda. Libo Kemkem Wereda is one of the wereda which found in South Gondar Zone of Amhara regional state. It is located90 kilometers far from Bahirdar (the capital city of Amhara Regional State) and it is 656 kilometers far from Addis Ababain the north direction. Addis zemen has a latitude and longitude of 12^o^07^’^37^o^47^’^E/12.117^o^N 37.783^o^E and an elevation of 1975 meters above sea level. The town is divided into three kebelles (the smallest unit of the woreda).

According to 2018 Adis Zemen town health statistics report, the estimated total population is 45, 125 of whom 22, 260 (49.3%) are men and 22, 865(50.7%) are women. The total number of women in the reproductive age group (15–49 years) is 14, 843 which accounts for 32.9% of the total town population. The town has one district hospital, one health center and two private clinics. Adis Zemen Hospital established in 2015 with a total of 91 staffs and currently, the hospital has a total of 236 staff[31].

### Participants

All pregnant women who attended antenatal clinics of the Adis Zemen Hospital were the source of population and all pregnant women who attended antenatal clinics of the Adis Zemen Hospital during the study period were the study population. All pregnant women who attended antenatal clinics of the Adis Zemen Hospital during the study period and who were voluntary to participate were included in the study whereas; all pregnant women who attended antenatal clinics of the Adis Zemen Hospital for the second time during the study period were excluded from the study.

### Sample size determination and sampling procedure

The required sample size was calculated using single population proportion formula;n = (Zα/2)^2^p(1-p)//d^2^ where; n is the required sample size, Z_a/2_ is the value of standard score at 95% confidence interval, p is the expected proportion of knowledge, and d^2^ is marginal error. And the following assumptions were used inorder to calculate the required sample size; 17.7% population proportion of malaria knowledge [25], 95% confidence interval, marginal error of 5% and 5% non-response rate. So the final sample size was 236 and those sampled participants were selected by systematic sampling technique. Since the data were collected for a one month period, the sampling interval was calculated by dividing the total number of client flows within one month by sample size. The average client flow for ANC clinic was 539 per month. Finally, the K^th^ value was found to be 2.3 (539/236) and every 2^nd^ woman was interviewed.

### Data collection tools and techniques

For the purpose of data collection, interviewer-administered questionnaire was adopted from differentliteratures. The questionnaire was prepared originally in English which had three parts like socio-demographic, and knowledge and utilization parts. The questionnaire was translated to the local language, Amharic for the purpose of data collection and it was translated back to English again for consistency. Before the actual data collection, pre-test was made on 5% of the total sample size of the respondent’s in Addis Zemen health centre. The data were collected via face to face interview by two diploma holder midwives under the guidance of one BSc midwife supervisor before the women receiving the care in waiting room. Two days of training about data collection procedures and research ethics was given for data collectors and supervisors.The data collection process was closely supervised on a daily basis and prompt feedback was given timely. Regular manual check-up for completeness and consistency was made.

### Variables and operational definition

The dependent variable of this study was women’s knowledge (poor/good knowledge) and the independent variables of this study were socio-demographic characteristics like age, religion, ethnicity, residence, occupation, marital status, monthly income, educational status, and means of communication.

**Knowledge on malaria;** was assessed by using 5 malaria knowledge related questions. Questions used to assess the knowledge were; 1) what is the causes of malaria?2) what are the sign and symptoms of malaria? 3) What is the mode of transmission of malaria? 4) what is the complication of malaria on pregnancy? 5) what are the prevention mechanism of malaria?. The first question (what is the causes of malaria?) had only 1 correct answer wheras the rest had multiples answer. Each multiples answer which was correct were considered as one point and coded 1 whereas incorrect answers were coded 0. Finally women’s knowledge on malaria was masured based on 22 points of 5 questions and dichotomized in to two;

**- Good Knowledge-** those who scored more than 60% of correct response for Knowledge related questions [24].

**- Poor Knowledge**—those who scored less than 60% of correct response for Knowledge related questions [24].

### Data analysis

The collected data were coded and entered into epidata software version 3.1 and exported to SPSS V-20 for analysis. The collected data were presented by frequency and percentage using tables, bar and pie charts. Mean and standard deviation was computed for numerical variables. To see the association between dependent and independent variables, binary and multivariate logistic regressions were used at 95% confidence interval. To control confounding factors, variable having a P value of<0.25 in binary logistic regression were transferred into multivariate logistic regression. After controlling confoundings, variables which had a P value of<0.05 were treated as predictor variables of knowledge. The direction and strength of association were determined based on adjusted odds ratio.

### Ethical considerations

The ethical clearance of this study was approved by an instituttional review board of Debre Tabor University. Before data collection, informed verbal consent was obtained from every respondent. Participants were informed about the purpose of study and their full right not to be interviewed at all or at any time. Participants were also informed that there was no direct benefit they gain in participating in this research. Confidentiality of participants was ensured through by keeping the information confidential, not including address and name of the respondents.

## Result

### Socio-demographic characteristics

From a total of the required 236 respondents, two hundred thirty-five mothers participated which made the response rate 99.6%. One hundred thirty-two mothers (56.2%) were in the age group of 25–34. All of the respondents (100%) were Amhara and the mean age of the participants was 28.1 years (SD ±4.8 years). One hundred sixty-six mothers (70.6%) were from urban and most of the participants (95.3%) were married. More than three-fourths of participants (81.3%) were orthodox Christian and around one-sixth of participants (15.7) were governmental employee. One fourth 59(25.1%) of participants couldn’t able to read and write. Most of participants 214 (91.1%) had at least one type of means of communication. Of them who had at least one type of communication, 211 (89.9%) of respondents had mobile Table 1.

**Table 1:** Socio-demographic characteristic of respondents in Addis zemen primary hospital, north- western, of Ethiopia 2018 (n = 235).

Variable	Category	Frequency	Percent (%)
Age	15–24	71	30.2
	25–34	132	56.2
	35–47	32	13.6
**Residence**	Urban	166	70.6
	Rural	69	29.4
**Marital status**	Married	224	95.3
	Separated	5	2.1
	Cohabited	4	1.7
	Single	2	0.9
**Religion**	Orthodox	191	81.3
	Muslim	35	14.9
	Protestant	9	3.8
**Occupation**	Housewife	100	42.6
	Governmental employee	37	15.7
	Merchant	98	41.7
**Education**	unable to read and write	59	25.1
	able to read and write	36	15.3
	primary education(1–8)	66	28.1
	secondary education(9–12)	24	10.2
	college or university	50	21.3
**Monthly income**	50 or less$	90	38.3
	51–100$	47	20
	101–150$	66	28.1
	151 or more$	32	13.6
**Having means of communication**	Yes	214	91.1
	No	21	8.9
**Types of means of communication**	Mobile	211	89.8
	Televesion	133	56.6
	Radio	75	31.9

### Malaria knowledge score

Of all a total of 235 subjects, 217 (91.6%) of the participants mentioned fever as a symptom of malaria and headache was mentioned by 183 (81%) of women. All of participants 235 (100%) said malaria can be transmitted through mosquito biting whereas 8 (3.4%) women said that malaria can be transmitted through direct contact. Two hundred four (86.8%) of participants listed abortion as a complication of malaria on pregnancy whereas, 156 (66.4%) of women listed stillbirth. When women asked to list the prevention mechanism of malaria all of the participants (100%) listed using ITN whereas, aminority of participants (1.3%) listed taking medicine as prevention mechanism of malaria Table 2. Overall women’s knowledge on malaria was measured based on correct response using five malaria knowledge questions and the question was scored out of 22 points. The minimum and maximum score of participants were 8 and 18 respectively. One hundred seventy-two (73.2%) of participants hadgood knowledge on malaria whereas, the rest 63 (26.8%) of participants had poor knowledge.

**Table 2:** Respondents knowledge on malaria in Addis zemen primary hospital, north- western, of Ethiopia 2018 (n = 235).

Variable	Category	Frequency	Percent (%)
**Cause of malaria**	Mosquito	235	100
	Fungus	0	0
	Virus	0	0
	Bacteria	0	0
**Sign and symptoms of malaria**	Headache	193	82.1
	Fever	217	92.3
	Shivering	210	89.4
	Back Pain	70	29.8
	Loss of Appetite	41	21.7
**Transmission of malaria**	Biting mosquito	235	100
	Drinking dirty water	13	5.5
	Exposed to Sun	5	2.1
	Exposed to cold air	21	6.4
	Direct contact	8	3.4
**Prevention mechanism of malaria**	Clean the house	168	71.5
	Using ITN	235	100
	Drainage of mosquito breeding sites	16	6.8
	Spray insecticide	43	22.6
	Clothing windows and doors at night	49	25.1
	Take medication	3	1.3
**Effect of malaria on pregnancy.**	Abortion	204	86.8
	Stillbirth	156	66.4
	Anemia	146	62.1
	Low birth weight	84	35.4
**Ever heard about malaria**	Yes	235	100
	Total	235	100

### Associated factors

The and multivariate logistic regression. First eight variables were tested in binary logistic regression. Variables which had a P value of <0.25 were transferred to multivariate logistic regression to control the confounding variables. Educational status, income, and residence were significantly associated with women’s knowledge on malaria. Women who were from urban were more knowledgeable than women who were from rural (AOR; 2.4: CI; 1.8, 5.7). Mothers who had family monthly income of 101–150 US dollars were more knowledgeable than mothers had family monthly income of 50 US dollars or less (AOR;3.4: CI; 2.7, 3.8). Participants who attended primary education were more knowledgeable than who could not able to read and write (AOR; 1.8: CI; 1.4, 3.5) Table 3.

**Table 3:** Factors associated with women’s knowledge on malaraia in Adis Zemen Hospital, North western Ethiopia, 2018(n = 235).

Variables	Knowledge		Crude Odd Ratio (95%CI)	Adjusted Odd Ratio (95%CI)
	Good	Poor		
**Age**				
≤30	132	49	1	1
>30	42	12	1.3(.5–2.8) *	1.1(.3–1.8)
**Residence**				
Urban	129	37	2.7(1.4–9.5)**	2.4 (1.8–5.7)**
Rural	39	30	1	1
**Marital status**				
Married	165	59	1	
Others	6	5	.4(.2–2.8)	
**Educational status**				
Unable to read and write	40	19	1	1
Able to read and write	20	16	.6(.2–1.4)	.4 (.2–1.5)
Primary education	52	14	2.6(1.3–5.9)**	1.8 (1.4–3.5)**
Secondary education	17	7	.98(.4–2.4) *	1.9 (.4–3.9)
College and above	45	5	4.2 (1.9–11.3)**	2. 3(1.9–4.8)**
**Occupation**				
Housewife	73	27	1	1
Governmental employee	34	3	4.2 (1.2–10.4)**	3.2 (.5–12.9)
Market trade vendor	67	31	.8(.4–1.5)	.6(.4–1.8)
**Income**				
50 or less$	55	35	1	1
51–100$	34	13	1.7(.8–3.6)	2.0(.3–7.8)
101–150$	58	8	4.6(2–6.7)**	3.4(2.7–3.8)**
151 or more$	27	5	3.4 (1.2–9.8)**	2.7(2.3–4.9)**
**Communication**				
Yes	157	57	1	
No	17	6	1.0(.4–2.1)	. 6 (.3–3.6)

NB

** indicates p-value<0.05, CI = confidence Interval.

* indicates p-value<0.25.

## Discussion

In this hospital-based cross-sectional study, we assessed women’s knowledge on malaria and associated factors among mothers attending antenatal clinics of Adis Zemen primary hospital and we found that 172 pregnant women (73.2%) had good knowledge on malaria. Assessing pregnant women’s knowledge on malaria and associated factors is very helpful for policymakers and stakeholders in planning maternal and child health care services. The figure of this study is lower than studies done in Nigeria (83.9%) [32]. This difference may be due to the fact that in the previous study all of the participants attended primary education and above. Educational status affects women’s knowledge so, the lower figure of women’s knowledge in the current study than the previous one may be due to this reason. This figure also lower than a study done in Cameroon (88%) [33]. This difference may be due to the fact that the study setting and sample size difference.

The finding of this study is comparable to studies done in Nigeria (71.5%) [21] and Ethiopia (73.4%) [24]. However, the finding of this study is higher than a study done in Nigeria, which showed that 64.9%[18] of the participants had good knowledge on malaria. This higher figure in the current the study may be due to the fact that the time variation between the two studies. The result of this study is also higher than studies done in Sudan (55.9%) [20]. This difference may be due to the study design and sampling procedure diffrenece which means in the current study, we have used hospital-based and probability sampling whereas in the previous Sudan study, they have used communitybased study design and non-probability sampling. This finding is also higher than a study done in Burkina Faso which revealed that 56.1% [19] of mothers had good knowledge. The difference may be due to the fact study population difference; in our study the study populations were pregnant women whereas in Burkina Faso study, the study populations were all reproductive age group women. This difference may be due to the fact that in the current study,we have used primary data whereas in previous study, they have used secondary data from Burkina Faso malaria indicator survey.

After controlling confounding factors in multivariate logistic regression; educational status, monthly income, and residence were found to be significantly associated with women’s knowledge on malaria.This finding revealed that living in the urban parts of the country increase the level of knowledge on malaria. Women who were from urban were 2.4 times more likely knowledgeable than women who were from rural. The association may be explained by women who are from urban may be more exposed for information like mass media and other health-related meeting than rural. This finding is supported by studies done in Nigeria [18] and Burkina Faso [19] which show that women from urban were more knowledgeable than rural. This result is also in line with a study done in Pawe, Ethiopia [34].

This finding indicated that monthly income was positively associated with women’s knowedge on malaria. Women who had family monthly income of 101–150 US dollars were 3.4 times more likely knowledgeable than mothers who had family monthly income of 50 US dollars or less. Mothers who had family monthly income of 151or more US dollars were2.7 times more likely knowledgeable than mothers had family monthly income of 50 US dollars or less. This association may be due to the fact that mothers who have better income may easily access information regarding malaria. In addition to this, the association may be due to the fact that women who have better income may visit health institutions while they are sick and may get information regarding to malaria. This is similar to studies done in Nigeria [21] and Ethiopia [34] as the studies revealed than mothers who had better family monthly income were more knowledgeable than mothers who had less family monthly income.

The other most important variable which was significantly associated with knowledge on malaria was women’s educational status. According to this study, educated women were more knwoledgeable than those who were not educated. Participants who attended primary education were 1.8 times more likely knowledgeable than who could not able to read and write respectively. Participants who attended college and above were 2.3 times more likely knowledgeable than who could not able to read and write.The finding of this study is similar to studies done inUganda [22], Cameroon [33], and Nigeria [18]. The finding of this study is also similar to studies done in Sudan [20]and Ethiopia [34]. The association may be due to that fact that educated mothers may easily read and understand information regarding malaria.

The current study was conducted among mothers who had antenatal visit and there might be a limitation of knowledge difference between women who had one ANC visit and woman who had three or four ANC visit. Since this study was a hospital based study design, the true figure of women’s knowledge on malaria might not be studied. Another limitation of this study was participants’ feeling about preconception care was not studied.

## Conclusion

Pregnant women’s knowledge on malaria is relatively high in Addis zemen hospital when it compares to most other similar studies. In this study, women’s educational status, income, and residence were predictor variables knowledge on malaria.This study confirmed that having a high educational status, having better income, being from urban lead to having good knowledge. Despite women’s knowledge is relatively high in Adis Zemen, increasing women’s knowledge about malaria via health education especially for those who are from rural, have no education, and have low month incomeis needed.

## Supporting information

S1 File Amharic version questionnaire. (DOCX)

S2 File English version questionaire. (DOCX)

## Abbreviations

ANC: Ante Natal Care
AOR: Adjusted Odds Ratio
CI: Confidence Interval
GDP: Gross Domestic Product
EDHS: Ethiopian Demographic Health Survey
ITN: Insecticide Bed Net
SD: standard Deviation
USA: United States of America
