# Supplementary material for: Malaria knowledge and its associated factors among pregnant women attending antenatal clinic of Adis Zemen Hospital, North-western Ethiopia, 2018
Source: PLoS One. 2019 Jan 10;14(1):e0210221. doi: 10.1371/journal.pone.0210221 (PMC6328161; doi:10.1371/journal.pone.0210221)
Supplement: S1 File — (DOCX) [file pone.0210221.s001.docx]

**በአማርኛ የተዘጋጀ መጠይቅ**

**ክፍል አንድ የማህበራዊ እና ስነህዝብ በተመለከተ የሚዳስሱ ጥያቄዎች**

| ተ.ቁ | ጥያቄ | መልስ |
| --- | --- | --- |
| 101 | ዕድሜዎት ስንት ነው? | በዓመት--------------- |
| 102 | የት ነው የሚኖሩት? | ሀ. ከተማ  ለ. ገጠር |
| 103 | የጋብቻ ሁኔታዎ ምንድን ነው? | ሀ. ያገባች  ለ. የፈታች  ሐ. የሞተባት  መ. ያላገባች  ሠ. ተለያይተው የሚኖሩ  ረ. ሳይጋቡ አበረው እሚኖሩ |
| 104 | ሀይማኖትዎ ምንድን ነው? | ሀ. ኦርቶዶክስ  ለ. ሙሰሊም  ሐ. ፕሮቴስታንት  መ. ሌላ (ይገለጥ) _____ |
| 105 | ብሄርዎ ምንድን ነው? | ሀ. አማራ  ለ. ኦሮሞ  ሐ. ትግራይ  መ. ሌላ (ይገለጥ) __________ |
| 106 | ስራዎ ምንድን ነው? | ሀ. የቤት እመቤት  ለ. የመንግስት ሰራተኛ  ሐ. ነጋዴ  መ.የቀንሰራተኛ  ሠ. ተማሪ  ረ. ሌላ (ይገለጥ)________ |
| 107 | የትምህርት ደረጃዎ ምንድን ነው? | ሀ. ማንበብ እና መፃፍ የማትችል  ለ. ማንበብ እና መፃፍ የምትችል  ሐ. አንደኛ ደረጃ (1-8)  መ. ሁለተኛ ደረጃ (9-12)  ሠ. ኮሌጅ ወይም ዩኒቨርስቲ |
| 108 | የቤቱ አማካይ ወርሀዊ ገቢ ስንት ነዉ? | ..............................በብር |
| 109 | የመገናኛ ዘዴዎች በቤትዎ ዉስጥ ይገኛሉ |  |
| 1010 | ለጥያቄ ቁጥር መልስዎ አዎ ከሆነ የትኞቹ የመገናኛ ዘዴዎች ናቸው በቤትዎ ዉስጥ ያሉት?  **(ብዙ መምረጥ ይቻላል)** | ሀ. ሬድዮለ. ቴሌቭዥን  ሐ. ሞባይል፣ስልክ መ. የለም |

**ለትብብርዎ እናመሰግናለን!**

**ክፍል 2 ስለ ወባ በሽታ ያለዎት ግንዛቤ በተመለከተ የሚዳስሱ ጥያቄዎች**

| 201 | አሁን በፊት ስለ አጎበር ሰምተሽ ታውቂአለሽ? | ሀ. አዎ  ለ. የለም |
| --- | --- | --- |
| 202 | የወባ በሽታን የሚያመጣው ረቂቅ ተዋህስያን ምንድን ነው? | ሀ. ትንኝ  ለ.ባክተሪያ  ሐ. ቫይረስ  መ. ፈንገስ |
| 203 | የወባ በሽታ ምልክቶች ምንምን ናቸው?  **(ብዙ መምረጥ ይቻላል)** | ሀ. ራስ ምታት  ለ የሰውነት ሙቀት  ሐ ማንቀጥቀጥ  መ ወገብህመም  ሠ የምግብ ፍላጎት መቀነስ  ረ. ሌላካለ, ይገለጥ-------- |
| 204 | የወባ በሽታ በምን በምን ይተላለፋል?  **(ብዙ መምረጥ ይቻላል)** | ሀ.በወባ ትንኝ በመነከስ  ለ. ቆሻሻ ውሀ በመጠጣት  ሐ. ጸሀይ ላይ በመስራት  መ. ለቀዝቃዛ አየር በመጋለጥ  ሠ. በንክኪ  ረ. ሌላካለ, ይገለጥ-------- |
| 205 | የወባ መከላከያ መንገዶች ምንምን ናቸው?  **(ብዙ መምረጥ ይቻላል)** | ሀ. ቤትን ንጹህ በማድረግ  ለ. ኬሚካል የተነከረ አጎበር በመጠቀም  ሐ የወባ ትንኝየሚራቡበትን ቦታ በማፋሰስ  ሠ. ጸረ ተባይ በመርጨት  ረ. ማታ ማታ በርና መስኮት በመዝጋት  ሰ. መድሀኒት በመጠቀም  ሸ. ሌላካለ, ይገለጥ-------- |
| 206 | የወባ በሽታ በእረግዝና ላይ ያለው ተጽእኖ ምንድን ነው?  **(ብዙ መምረጥ ይቻላል)** | ሀ. ውርጃ  ለ. ሙቶ መወለድ  ሐ. የደም ማነስ  መ. በክብደት ትንሽ ልጅ መውለድ  ሠ. ሌላካለ, ይገለጥ-------- |

**ስለትብብርዎ ከልብ እናመሰግናለን!!!!!!!!!!**
