# Supplementary material for: Malaria knowledge and its associated factors among pregnant women attending antenatal clinic of Adis Zemen Hospital, North-western Ethiopia, 2018
Source: PLoS One. 2019 Jan 10;14(1):e0210221. doi: 10.1371/journal.pone.0210221 (PMC6328161; doi:10.1371/journal.pone.0210221)
Supplement: S2 File — (DOCX) [file pone.0210221.s002.docx]

**English version questionnaire**

**Part I: Socio -demographic factors**

| Sr. No | **Questions** | Answer to questions |
| --- | --- | --- |
|  | What is your age? | ................. in years |
|  | What is your residence? | A .urban  B . rural |
|  | What is your marital status? | A. Married  B. Divorced  C. Widowed  D. Single  E. Separated  F. Cohabited |
|  | What is your religion? | A. Orthodox  B. Muslim  C. Protestant  D. Others(specify) ____________ |
|  | What is your ethnicity? | A. Amhara,  B. Oromo  C. Tgray  D. Others, specify---------- |
|  | What is your occupation? | A. Farmer  B. House wife  C. Governmental Employee  D. Merchant  E. Daily Labourer  F. Student  G. Other specify....... |
|  | What is your educational Status? | A. un able to read and write  B. able to read and write  C. Primary education(1-8)  D. Secondary education(9-12)  E. College or University |
|  | What is your monthly household income? | .................in birr |
|  | Do you have any of the following means of communication? | 1. Yes 2. No |
|  | If yes for question number 109, which types of means of communication do you have?  Multiples answers are possible | A. Radio  B. TV  C. mobile  D. None |

**Part II: Questions related to knowledge on malaria**

| 201 | Have you ever heard about malaria? | 1. Yes 2. No |
| --- | --- | --- |
| 202 | What is the cause of malaria? | 1. Mosquito 2. Bacteria 3. Virus 4. Fungus |
| 203 | What are the sign and symptoms of malaria?  Multiples answers are possible | 1. Headache 2. Fever 3. Chills 4. Shivering 5. Loss of appetite 6. Back pain 7. Others |
| 204 | What is the transmission of malaria? Multiples answers are possible | 1. By mosquito biting 2. Drinking dirty water 3. Working in the sun 4. Exposure to cold air 5. Direct contact 6. Others |
| 205 | What are the prevention mechanisms of malaria? Multiples answers are possible | 1. AKeep house clean 2. Insecticide treated bed nets 3. Drainage of mosquito breeding sites 4. Spray insecticide 5. Clothing windows and doors at night 6. Take medicine 7. Others |
| 206 | What is the effect of malaria on pregnancy? Multiples answers are possible | 1. Abortion 2. Still birth 3. Anemia 4. Low birth weight 5. Others |

**Thank you for your cooperation!!!!!**
